# Chemsex among men who have sex with men living outside major cities and associations with sexually transmitted infections: A cross-sectional study in the Netherlands

**DOI:** 10.1371/journal.pone.0216732

**Published:** 2019-05-14

**Authors:** Ymke J. Evers, Geneviève A. F. S. Van Liere, Christian J. P. A. Hoebe, Nicole H. T. M. Dukers-Muijrers

**Affiliations:** 1 Department of Sexual Health, Infectious Diseases and Environmental Health, Public Health Service South Limburg, Heerlen, The Netherlands; 2 Department of Medical Microbiology, Care and Public Health Research Institute (CAPHRI), Maastricht University Medical Center, Maastricht, The Netherlands; Agencia de Salut Publica de Barcelona, SPAIN

## Abstract

**Background:**

The intentional use of drugs to have sex–chemsex–among men who have sex with men (MSM) might contribute to the high sexually transmitted infections (STI) prevalence in this group. Limited data is available on chemsex outside major cities. The current study investigated the use of a wide variety of drugs during sex among MSM living outside major cities in the Netherlands and their associations with STI.

**Methods:**

In 2018, 350 MSM were recruited at STI clinics and 250 MSM completed an online questionnaire. Questionnaire data were linked to clients’ most recent STI laboratory test results. Chemsex was defined as using cocaine, crystal meth, designer drugs, GHB/GBL, ketamine, speed, or XTC/MDMA during sex in the preceding six months. The use of other drugs was also assessed. Determinants (chemsex, specific drugs, number of drugs, combining, and frequency) potentially associated with STI were assessed using multivariable logistic regression analyses adjusting for sociodemographic characteristics and sexual history.

**Results:**

Chemsex was reported by 35% (95%CI: 29–41) of the 250 participants. XTC/MDMA (27%; 68/250) and GHB/GBL (26%; 64/250) were the most used drugs. STI positivity was 33% (29/87) in MSM engaging in chemsex and 12% (12/163) in MSM not engaging in chemsex (p<0.001). Half of MSM engaging in chemsex (45/87) used three of more different chemsex drugs; STI positivity in this group was 44% (20/45). The only factor independently associated with STI was the use of three or more chemsex drugs (aOR: 4.13, 95%CI: 1.77–9.62).

**Conclusion:**

This study shows that chemsex is prevalent among MSM visiting the STI clinic outside major cities in the Netherlands, suggesting that health services in both urban and non-urban areas should be aware of and informed on chemsex. MSM who used multiple drugs are at particular risk for STI, indicating a special need for STI prevention and care in this group.

## Introduction

Men who have sex with men (MSM) are an important risk group for the on-going transmission of sexually transmitted infections (STI), including human immunodeficiency virus (HIV), in many industrialized countries [1–3]. In the Netherlands, more than half of new *Neisseria gonorrhoeae* and HIV infections and almost all syphilis infections occur among MSM [4]. Studies have showed that drug use during sex, or ‘chemsex’, is prevalent among MSM [5–9] and this might contribute to the burden of STI in this group. Chemsex has been described as the practice of intentionally using drugs before or during sex to enable, enhance and prolong sexual interactions. Crystal methamphetamine (crystal meth), gamma-hydroxybutyric acid (GHB)/gamma-butyrolactone (GBL), and mephedrone have been described as typical ‘chemsex drugs’ by research in the United Kingdom (UK) [10]. Drug use can have an impact on decision-making processes whereby an individual may engage in certain risk behaviours that were not intended prior to the consumption of drugs [11]. A recent systematic review [12] identified seventeen studies from Europe, Australia and the United States of America (USA) that showed an increased risk of condomless anal sex in MSM using the chemsex drugs. Several studies identified that chemsex was also associated with a higher likelihood of esoteric sex acts (e.g. group sex, prolonged sex sessions, and fisting) [8, 13–15]. Aside from sexual risk behaviour, chemsex has been associated with risky drug administration methods, such as injecting drugs and sharing injecting equipment [8]. These risk behaviours make MSM engaging in chemsex more prone to STI transmission. Indeed, use of the chemsex drugs has been associated with an increased risk for STI in several studies [8, 16, 17].

Recent studies in the UK indicated that chemsex should be addressed as a public health priority [16, 18, 19]. The European MSM Internet Survey (EMIS) in major cities in 44 European countries showed that the use of chemsex drugs was also high in Amsterdam [20], suggesting that chemsex might also be a public health issue in the Netherlands [21]. A recent study confirms that chemsex drugs are frequently used by MSM in Amsterdam [17]. It is likely that chemsex might also be prevalent outside major cities [22]. However, limited epidemiological data on chemsex and the specific drugs used during sex is available outside major cities. Knowledge on this issue outside major cities is needed to enable STI services in non-urban areas to plan for possible increasing needs of their MSM clients. Moreover, most studies are currently focused on the use of the three chemsex drugs and its association with STI [8, 16, 17, 20]. By using this narrow definition of chemsex, STI risks related to the use of other stimulants and depressants during sex might be missed in research and be insufficiently addressed in STI prevention strategies and clinical practice. Rapidly changing drug trends and the current rise in newly reported psychoactive substances in Europe [23] indicate a need to broaden the assessment of drugs used during sex and their association with STI. To address these knowledge gaps, the aim of this study was twofold: assessing (1) the prevalence of chemsex and a wide variety of drugs used during sex outside major cities, and (2) its associations with STI.

## Methods and materials

### Ethics statement

This study was approved by the Medical Ethical Committee of the University of Maastricht (METC 2018–0485). STI client registry data was collected within standard care. During recruitment, STI nurses provided information on the study and participants gave oral informed consent. Data from questionnaires and linkage to data from medical records were retrieved in a coded de-identified manner which was untraceable to participants by the researchers.

### Data collection

The outpatient Public Health Service STI clinics Limburg in the Netherlands (9000 consultations annually) offer free and anonymous STI and HIV testing, hepatitis B vaccinations, and sexual health counselling to risk groups, among which are MSM. Limburg, a southern area in the Netherlands, consists of both urban (≥1500 addresses/m^2^) and non-urban areas (<1500 addresses/m^2^), according to the definition of Statistics Netherlands (www.cbs.nl). For this study, MSM aged 16 years or older who attended one of the STI clinics in Limburg were asked to fill in an online questionnaire on drug use. MSM were defined as men who had sex with men in the past six months. Convenience sampling was used for the recruitment Questionnaires were available in both Dutch and English. The recruitment period was from September, 2017, till June, 2018. After the consultation, the questionnaire was sent by email or text message to all MSM who had agreed to receive the questionnaire. Duplicate survey entries were not possible, as we used an online survey program that remembered the questions a participant already completed. Forwarding was possible but highly unlikely as participants were invited personally and consented for participation.

#### Client registry data

All MSM were routinely universally tested for urogenital, anorectal, and oropharyngeal *Chlamydia trachomatis* (CT) and *Neisseria gonorrhoeae* (NG), HIV, hepatitis B and syphilis. Specimens tested for CT and NG consisted of urine, self-collected anorectal swabs and nurse-collected oropharyngeal swabs. Specimens were processed at the regional medical microbiology laboratory of Maastricht University Medical Centre using polymerase chain reaction (PCR) (Cobas 4800, Roche, California). Serum was tested for HIV (anti-HIV[1/2]) Hepatitis B (HBsAg II and Anti-HBs), and syphilis (*Treponema pallidum*, Biokit 3.0). Each consultation further included a standardized nurse-taken medical and sexual history, including sociodemographic characteristics, symptoms, previous STI diagnosis, and number of sex partners in the preceding six months. All data were registered in an electronic client registry using a consultation code. Electronic client registry data of the most recent consultation during the research period was linked to the online questionnaire data using the consultation code.

#### Questionnaire

All participants were asked if they had used drugs before or during sex in the preceding six months and if so which ones (list of drugs asked can be found in S1 File) and how frequent they used these drugs (≥4 times per week, 2–4 times per week, ≤1 time per month, not in preceding six months). Slang names of all drugs were included in the questionnaire. In those reporting any drug use during sex, additional questions were asked on sober sex (sex without the use of any drug), combining drugs during sex, combining alcohol and drugs during sex, sexual risk behaviours while under the influence of drugs, injection of drugs (‘slamming’), anal insertion of drugs with syringes (‘booty bumping’), sharing needles, syringes or snort tubes (questions can be found in S2 File).

### Definitions

The outcome measure was an STI diagnosis, this was defined as being diagnosed with CT, NG, infectious syphilis (Lues I, Lues II, Lues latens recens), acute hepatitis B and/or a newly diagnosed HIV infection at the most recent consultation of the client during the research period.

The main determinant of interest was drug use during sex, and three different definitions were used. Any drug use during sex was defined as the use of any of the drugs listed in S1 File during sex in the preceding six months. We defined chemsex as the use of crystal meth, cocaine, designer drugs (2-CB, 3 MMC, 4-FA, 4-MEC), GHB/GBL, ketamine, mephedrone, speed, or ecstasy/3,4-Methyl enedioxy methamphetamine (XTC/MDMA) during sex in the preceding six months (referred to as broad definition chemsex). Cannabis, poppers, laughing gas and magic mushrooms are generally excluded from the chemsex definition because of their use in a broader context [19]. We also constructed the UK definition of chemsex [10] and this included the typical chemsex drugs: the use of crystal meth, GHB/GBL and/or mephedrone during sex in the preceding six months. The drugs of the broad definition of chemsex were used to construct the following measures. Frequency of chemsex was calculated from the drug that was most frequently used and categorized into never, ≤1 time per month, 2–4 times per month, ≥2 times per month. The number of different drugs used was calculated as the sum of drugs used, and categorized into 0 drugs, 1–2 drugs, ≥3 drugs. Combining drugs was defined as the use of at least two different drugs and combining drugs and alcohol was defined as the use of drugs and at least four units of alcohol during sex.

### Statistical analysis

A non-participation analysis was performed, in which sociodemographic characteristics and STI positivity were compared between participants and non-participants using using χ^2^ tests. Participants were defined as all MSM who fully completed the questionnaire, and non-participants were defined as all MSM who visited the STI clinic in the study period and were not asked, did not agree to participate, or did not complete the questionnaire.

Descriptive statistics were used to describe proportion of MSM engaging in chemsex, the specific drugs used, number of drugs used, frequency of chemsex, and combining different drugs. For the measures of any drug use, chemsex and use of the typical chemsex drugs, 95% confidence intervals (CI) were calculated. Sociodemographic characteristics and STI risk factors were compared between MSM who tested STI negative and MSM who tested STI positive, using chi-square tests and Fisher exact tests. STI positivity was presented for all categories of the characteristics. The characteristics that showed statistically significant differences were included as possible confounders in further analyses. We assessed associations between a range of chemsex-related behaviours and STI using univariable logistic regression analyses. Factors included any drug use, broad definition of chemsex, UK definition of chemsex, use of specific drugs during sex, frequency of chemsex, number of drugs used, combining drugs, combining alcohol and drugs, use of erectile dysfunction drugs and use of tranquilizers. Associations between STI and all factors were adjusted for significant confounders in separatemultivariable models. All factors with a p-value <0.05 after adjustment for confounders were included in one multivariable model. The model was adjusted for significant confounders. We used a forward regression model, because of multicollinearity between several determinants (Spearman’s correlation coefficient ≥ 0.8), such as frequency of chemsex and number of drugs used. Additional analyses were restricted to participants engaging in chemsex. Descriptive analyses were used to assess the proportion of MSM engaging in risky drug administration and sexual risk behaviour. All analyses were performed using SPSS V21 (IBM SPSS Statistics for Windows, IBM Cooperation, Armonk, New York, USA).

## Results

### Study population

**Fig 1:**
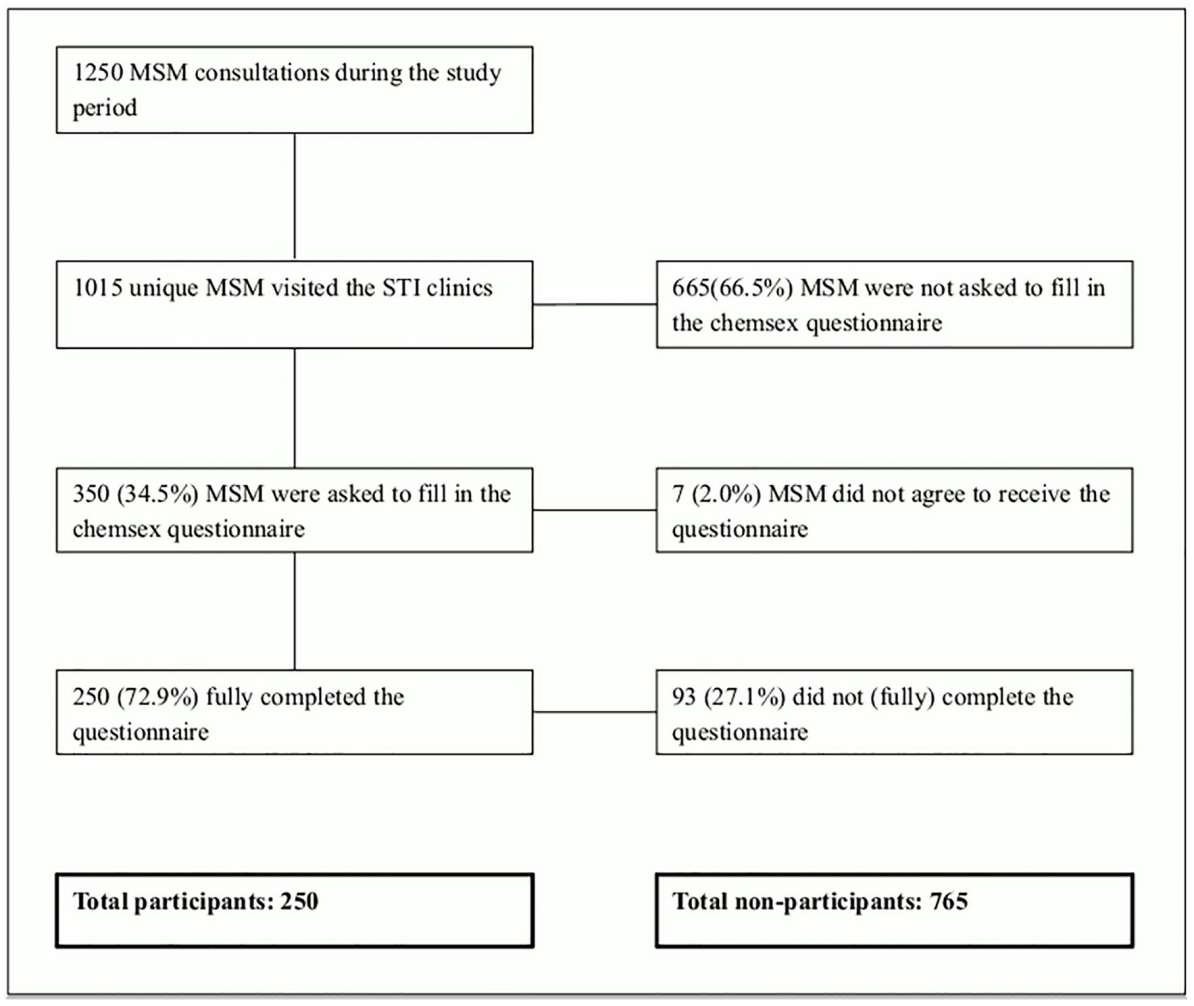
Flow chart of study population.

Of the 1015 unique MSM attending the STI clinics during the study period, 350 (34.5%) were recruited to participate, and 250 (72.9%) fully completed the questionnaire (Fig 1). Age (median age 33 years versus median age 34 years, p = 0.75), educational level (higher educated: 64% versus 60%, p = 0.30), ethnicity (western: 91% versus 88%, p = 0.12), and STI positivity (19% versus 18%, p = 0.53) were comparable between MSM who were recruited and MSM who were not recruited. Age (median age 35 years versus median age 31 years, p = 0.07), ethnicity (western: 93% versus 86%, p = 0.08), and STI positivity (19% versus 19%, p = 0.97) were comparable between who completed the questionnaire and MSM who were recruited but not completed the questionnaire. MSM who completed the questionnaire were more often higher educated (67% versus 54%, p = 0.05) than MSM who were recruited but not completed the questionnaire. In total, age (median age 35 years versus median age 33 years, p = 0.50), educational level (higher educated: 67% versus 60%, p = 0.06), and STI positivity (19% versus 18%, p = 0.57) were comparable between participants (N = 250) and non-participants (N = 765). Participants more often had a western nationality compared to non-participants (93% versus 88%, p = 0.03).

### Drug use during sex

**Fig 2:**
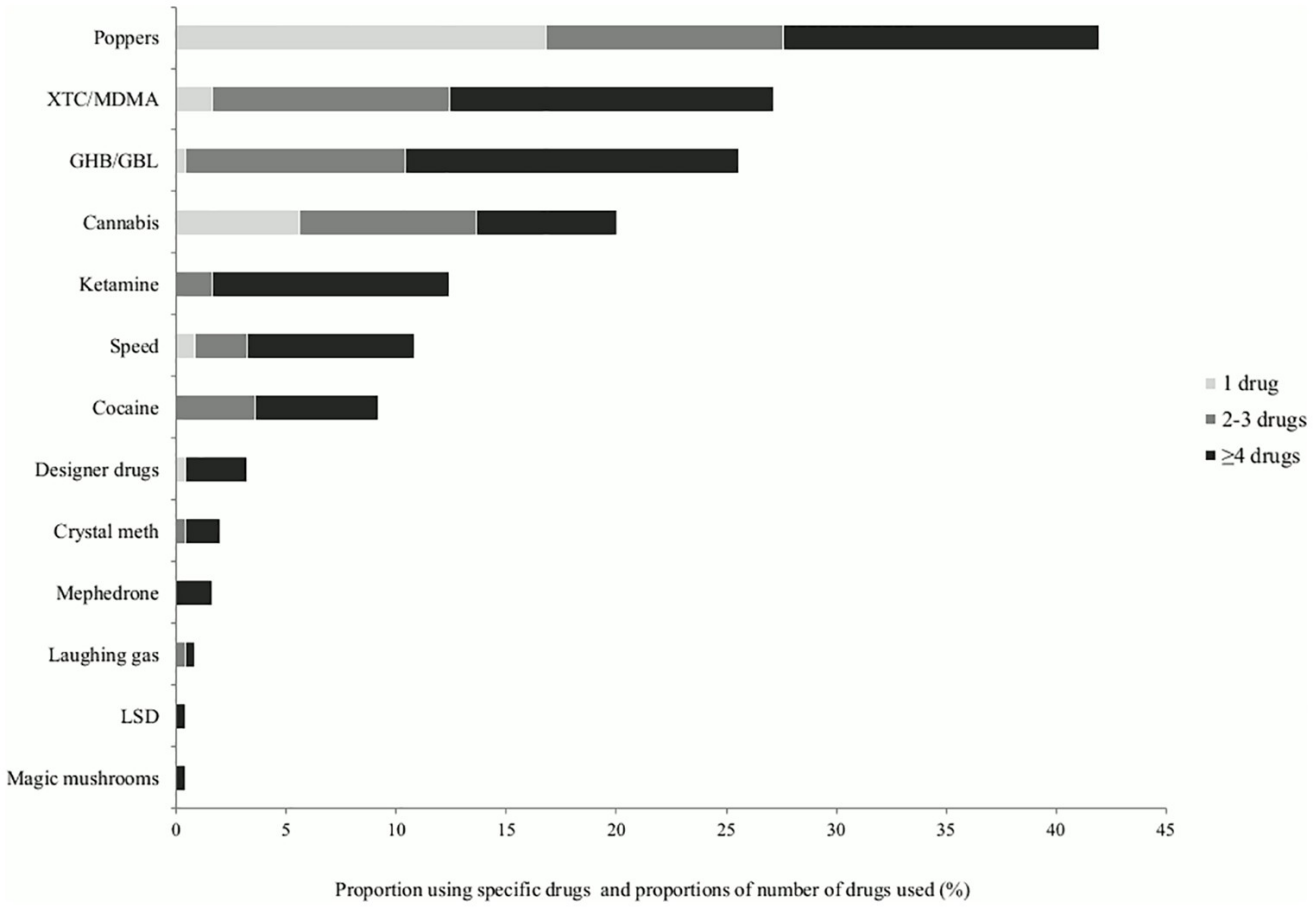
Drug use in the preceding six months in all participants (N = 250).

Any drug use during sex was reported by 54% (95%CI: 48–61) of the 250 participants. Poppers (42%; 105/250), XTC/MDMA (27%; 68/250) and GHB/GBL (26%; 64/250) were the most used drugs during sex. Of MSM using at least one drug during sex, 29% (40/136) used four or more different drugs in the preceding six months. Of MSM using poppers, 40% used only this drug. Of MSM using XTC/MDMA, GHB/GBL, ketamine, speed, cocaine, crystal meth or mephedrone, 93–100% used at least one other drug (Fig 2). One of four participants combined different drugs during one chemsex session (60/250), with XTC/MDMA and GHB/GBL (78%; 47/60) being the most used combination. Among MSM using ≥4 drugs, 98% (39/40) combined different drugs at one sex session.

Chemsex (cocaine, crystal meth, designer drugs, GHB/GBL, ketamine, mephedrone, speed, XTC/MDMA) was reported by 35% (87/250) (95%CI: 29–41); the proportion of chemsex was comparable between MSM living in a urban-area and MSM living in a non-urban area (36% vs. 33%; p = 0.68). Of MSM engaging in chemsex, 49% (43/87) engaged in chemsex in the past week. Sober sex in the past month was reported by 72% (63/87). The use of at least one of the typical chemsex drugs was reported by 26% (65/250) (95%CI: 21–32), of which GHB/GBL was the most used drug (99%; 64/65). Crystal meth (2%; 5/250) and mephedrone (2%; 4/250) were rarely reported.

### STI positivity

In total, 19% (48/250) of all participants were tested positive for an STI. Positivity was 10% (24/250) for CT, 9% (23/250) for NG, 2% (4/250) for infectious syphilis, 1% (2/250) for HIV. STI positive and STI negative tested men differed regarding age, HIV status, and number of sex partners. In other words, STI positivity was higher in older MSM, known HIV positive MSM, and MSM who had seven or more sex partners in the preceding six months (Table 1).

**Table 1:** Characteristics of study population and STI positivity within groups.

	All participants (N = 250)	STI positive (N = 48)	STI negative (N = 202)	
	% of total (N)	% within groups (N)	% within groups (N)	P (χ^2-^test)
***Sociodemographic characteristics***				
**Age in years**^a^				**0.007**
16–28	27.6 (69)	17.4 (12)	82.6 (57)	
29–43	36.8 (92)	10.9 (10)	89.1 (82)	
44–71	35.6 (89)	29.2 (26)	70.8 (63)	
**Educational level**^b^				0.963
Lower educated	33.0 (76)	19.7 (15)	80.3 (61)	
Higher educated	67.0 (154)	19.5 (30)	80.5 (124)	
**SES**^a^^,^^c^				0.507
Low	30.8 (77)	22.1 (17)	77.9 (60)	
Medium	33.8 (79)	15.2 (12)	84.8 (67)	
High	31.2 (78)	16.7 (13)	83.3 (65)	
**Urbanization**^c^				0.854
Non-urban	45.6 (114)	18.4 (21)	81.6 (93)	
Urban	48.0 (120)	17.5 (21)	82.5 (99)	
**Ethnicity**^b^				0.740
Western	92.8 (232)	19.0 (44)	81.0 (188)	
Non-western	7.2 (18)	22.2 (4)	77.8 (14)	
**Sexual identity**				0.636
Homosexual	87.6 (219)	19.6 (43)	80.4 (176)	
Bisexual	12.4 (31)	16.1 (5)	83.9 (26)	
***Sexual history***				
**Commercial sex work**				0.576^d^
Yes	1.6 (4)	25.0 (1)	75.0 (3)	
No	98.4 (246)	19.1 (47)	80.9 (199)	
**HIV status**^e^				**0.011**
Aware positive	19.6 (49)	32.7 (16)	67.3 (33)	
Negative or unknown	80.4 (201)	15.9 (32)	84.1 (169)	
**Previous STI diagnosis**				0.123
Yes	24.8 (62)	18.4 (21)	81.6 (41)	
No	45.6 (114)	27.4 (17)	72.6 (97)	
Unknown	29.6 (74)	13.5 (10)	86.5 (64)	
**Notified for STI**				0.262
Yes	16.8 (42)	26.2 (11)	73.8 (31)	
No	83.2 (208)	18.0 (37)	82.0 (166)	
**Number of sex partners <6 months**^a^				**0.014**
1–3	32.4 (81)	11.1 (9)	88.9 (72)	
4.7	30.8 (77)	16.9 (13)	83.1 (64)	
≥7	36.8 (92)	28.3 (26)	71.7 (66)	

Row percentages are presented.

^a^ Based on tertile distributions.

^b^ Educational level and ethnicity were based on the definitions used by the Statistics Netherlands (www.cbs.nl).

Western: person who was born in Europe (excluding Turkey), North-America, Oceania, Indonesia or Japan.

Non-Western: person who was born or of whom at least parent was born in Africa, Latin America, Asia (excluding Indonesia or Japan) or Turkey.

^c^ Postal codes were used to calculate socioeconomic status (SES) (www.//scp.nl) and urbanization (www.cbs.nl). Urbanization was categorized into urban (≥1500 addresses/m^2^) and non-urban (<1500 addresses/m^2^). Data of 16 MSM were missing as postal code was not recorded.

^d^ Fisher-exact test

^e^ HIV status included prior HIV diagnoses only and excluded newly diagnosed HIV diagnoses.

### STI and associated determinants

MSM engaging in chemsex more often were STI positive than MSM not engaging in chemsex (33% vs. 12%; p<0.001). The association between chemsex and STI was similar for MSM living in urban areas and MSM living in non-urban areas (data not shown). The association between chemsex and STI remained significant after adjustment for age, HIV status and number of sex partners in the preceding six months (aOR: 2.92, 96%CI: 1.42–6.01). Likewise, the use of XTC/MDMA, GHB/GBL, and cocaine were significantly associated with STI after adjustment for confounders (Table 2). However, the only variable associated with STI in the adjusted multivariable model was the use of three or more chemsex drugs in the preceding six months (aOR: 4.13; 95%CI: 1.77–9.62). STI positivity was 44% (20/45) in MSM who used three or more drugs, compared to 21% (9/42) in MSM who used one or two drugs, and 12% (19/163) in MSM who did not use any drug.

**Table 2:** Percentages of chemsex and drugs used during sex and associations with STI positivity in MSM attending an STI clinic.

	All participants (N = 250)	STI positive (N = 48)	STI negative (N = 202)	Outcome: STI positivity	
	% of total (N)	% within groups (N)	% within groups (N)	Unadjusted OR (95% CI)	Adjusted OR (95% CI)^a^
***Main behaviours***					
**Any drug use during sex**					
Yes	54.4 (136)	23.5 (32)	76.5 (104)	1.89 (0.97–3.65)	1.36 (0.67–2.79)
No	45.6 (114)	14.0 (16)	86.0 (98)	1^b^	1
**Broad definition chemsex**^c^					
Yes	34.8 (87)	33.3 (29)	66.7 (58)	3.79 (1.97–7.29)***	2.92 (1.42–6.01)***
No	65.2 (163)	11.7 (19)	88.3 (144)	1	1
**UK definition chemsex**					
Yes	26.0 (65)	36.9 (24)	63.1 (41)	3.93 (2.03–7.61)***	2.87 (1.39–5.97)**
No	74.0 (185)	13.0 (24)	87.0 (161)	1	
***Chemsex-related behaviour based on broad definition of chemsex***					
**Number of different chemsex drugs used <6 months**					
≥3	18.0 (45)	44.4 (20)	55.6 (25)	6.06 (2.84–12.94)***	4.13 (1.77–9.62)**
1–2	16.8 (42)	21.4 (9)	78.6 (33)	2.07 (0.86–4.98)	1.92 (0.76–4.87)
0	65.2 (163)	11.7 (19)	88.3 (114)	1	1
**Combining chemsex drugs in one sex session**					
Yes	24.0 (60)	31.7 (19)	68.3 (41)	2.57 (1.31–5.04)*	1.70 (0.81–3.58)
No	76.0 (190)	15.3 (29)	84.7 (161)	1	1
**Combining drugs and alcohol (≥4 units) during one sex session**					
Yes	20.4 (51)	15.7 (8)	84.3 (43)	0.74 (0.32–1.70)	0.47 (0.18–1.21)
No	79.1 (199)	20.1 (40)	79.1 (159)	1	1
**Frequency chemsex <6 months**					
Twice per month or more	16.0 (40)	45.0 (18)	55.0 (22)	6.20 (2.83–13.60)***	**4.23 (1.78–10.05)**
Once per month or less	18.8 (47)	23.4 (11)	76.6 (36)	2.32 (1.01–5.30)*	1.99 (0.81–4.91)
Never	65.2 (163)	11.7 (19)	88.3 (144)	1	1
***Specific drugs used during sex***					
**Poppers**					
Yes	42.0 (105)	26.7 (28)	73.3 (77)	2.27 (1.20–4.31)*	1.71 (0.86–3.40)
No	58.0 (145)	13.8 (20)	86.2 (125)	1	1
**XTC/MDMA**					
Yes	27.2 (68)	35.3 (24)	64.7 (44)	3.59 (1.86–6.93)***	2.67 (1.29–5.56)**
No	72.8 (182)	13.2 (24)	86.8 (158)	1	1
**GHB/GBL**					
Yes	25.6 (64)	35.9 (23)	64.1 (41)	3.61 (1.81–7.00)***	2.59 (1.24–5.24)*
No	74.4 (186)	13.4 (25)	86.6 (161)	1	1
**Cannabis**					
Yes	22.8 (57)	22.8 (13)	77.2 (44)	1.33 (0.65–2.74)	1.03 (0.47–2.22)
No	77.2 (193)	18.1 (35)	81.9 (158)	1	1
**Ketamine**					
Yes	12.4 (31)	35.5 (11)	64.5 (20)	2.71 (1.20–6.12)*	1.78 (0.74–4.25)
No	87.6 (219)	16.9 (37)	83.1 (182)	1	
**Speed**					
Yes	10.8 (27)	25.9 (7)	74.1 (20)	1.55 (0.62–3.92)	1.17 (0.44–3.11)
No	89.2 (223)	18.4 (41)	81.6 (182)	1	1
**Cocaine**					
Yes	9.2 (23)	47.8 (11)	52.2 (12)	4.71 (1.93–11.47)**	3.01 (1.12–8.05)*
No	90.8 (227)	16.3 (37)	83.7 (190)	1	1
**Designer drugs**					
Yes	3.2 (8)	37.5 (3)	62.5 (5)	NA^d^	NA
No	96.8 (242)	18.6 (45)	81.4 (197)		
**Crystal meth**					
Yes	2.0 (5)	20.0 (1)	80.0 (4)	NA	NA
No	98.0 (245)	19.2 (47)	80.2 (198)		
**Mephedrone**					
Yes	1.6 (4)	50.0 (2)	50.0 (20)	NA	NA
No	98.4 (246)	18.7 (46)	81.3 (200)		
**Nitrous oxide**					
Yes	0.8 (2)	0.0 (0)	100.0 (2)	NA	NA
No	99.2 (248)	19.4 (48)	80.6 (200)		
**LSD**					
Yes	0.4 (1)	100.0 (1)	0.0 (0)	NA	NA
No	99.6 (249)	18.9 (47)	91.1 (202)		
**Magic mushrooms**					
Yes	0.4 (1)	100.0 (1)	0.0 (0)	NA	NA
No	99.6 (249)	18.9 (47)	91.1 (102)		
***Substance use during sex***					
**Erection dysfunction drugs**					
Yes	41.6 (104)	30.8 (32)	69.2 (72)	3.61 (1.86–7.03)***	2.63 (1.26–5.47)*
No	58.4 (146)	11.0 (16)	89.0 (130)	1	1
**Tranquilizers**					
Yes	8.8 (22)	18.2 (4)	81.8 (13)	0.93 (0.30–2.88)	0.83 (0.25–2.73)
No	91.2 (228)	19.3 (44)	80.7 (184)	1	1

*** <0.001,

** <0.01,

*<0.05

Row percentages are presented.

The use of basecoke, heroin and ritalin during sex in the preceding six months was reported by none of the participants.

^a^ Adjusted for age, HIV status, and number of sex partners in the preceding six months (based on Table 1).

^b^ The reference category is indicated with value ‘1’.

^c^ Drugs included in the broad definition of chemsex: cocaine, crystal meth, designer drugs, GHB/GBL, ketamine, mephedrone, speed, XTC/MDMA.

^d^ NA: groups were too small to calculate odds ratios.

### Sexual risk behaviour and risky drug administration among MSM engaging in chemsex

Condomless receptive anal sex was reported by 64% (56/87) and 40% (34/87) engaged in one chemsex session that lasted for at least 12 hours. Slamming was reported by 6% (5/87) and none of the participants reported to have shared needles. Other percentages of sexual risk behaviours and risky drug administration methods can be found in Table 3.

**Table 3:** Sexual risk behaviour and risky drug administration in the preceding six months among MSM engaging in chemsex (N = 87).

	% (N)
**Sexual risk behaviour**	
Condomless receptive anal sex	64.4 (56)
Condomless insertive anal sex	59.8 (52)
Condomless receptive anal sex with play toys	35.6 (31)
Chemsex session lasting ≥12 hours	39.1 (34)
Being fisted	20.7 (18)
Multiple sex partners at one chemsex session	36.8 (32)
Casual sex partner(s) (name unknown)	21.8 (19)
**Risky drug administration**	
Slamming	5.7 (5)
Sharing needles	0.0 (0)
Booty bumping	12.6 (11)
Sharing syringes	4.6 (4)
Sharing snorting tubes	37.9 (33)

## Discussion

Our study shows that more than half of MSM who visited the STI clinic in outside major cities in the Netherlands used any drug during sex in the preceding six months. Poppers, XTC/MDMA, and GHB/GBL were the most used drugs. When using the broad definition of chemsex (the use of crystal meth, cocaine, designer drugs, GHB, ketamine, mephedrone, speed and/or XTC/MDMA during sex), chemsex was reported by 35% (95%CI: 29–41). The proportion of MSM engaging in chemsex was comparable between men who lived in urban areas and men who lived in non-urban areas. More than ninety percent of men using XTC/MDMA, GHB/GBL, ketamine, speed, cocaine, designer drugs, crystal meth, or mephedrone engaged in polydrug use (using other drugs as well). We demonstrated a strong and consistent association between the use of three or more of these drugs and an increased risk for STI. This study informs sexual health clinics outside major cities in the Netherlands on emerging drug trends and their association with STI and hereby helps them to anticipate the needs of their clients.

With regard to the prevalence of chemsex outside the major cities, our data shows that the three typical chemsex drugs–crystal meth, mephedrone, GHB/GBL—are used by 26% of MSM visiting the STI clinic outside the major cities in the Netherlands. It should be noted that this percentage mainly reflects the use of GHB/GBL. When comparing our results on the use of these drugs in the preceding six months to data in MSM from the STI clinic in Amsterdam[17], we demonstrated higher use of GHB in our study (26% vs. 16%), but similar use of crystal meth (2% vs. 4%) and mephedrone (2% vs. 3%). The prevalence of slamming was comparable low in both studies (2% vs. 1%). Our results show that the use of other drugs, especially XTC/MDMA, ketamine, speed and cocaine, during sex was also prevalent and combining different drugs was common. Almost all men using one of the drugs of the broad definition of chemsex (crystal meth, cocaine, designer drugs, GHB/GBL, ketamine, mephedrone, speed, XTC/MDMA) used more than one drug in the preceding six months. Our study suggests that drug use during sex is prevalent among MSM visiting the STI clinic outside major cities in the Netherlands and that health services in non-urban areas should be aware of this phenomenon. Specifically, the high prevalence of polydrug use indicates that drug prevention and harm reduction strategies targeted at MSM engaging in chemsex should include the risks of other drugs next the typical chemsex drugs and interactions between different drugs (such as the increased risk of an overdose and coma when combining for example GHB/GBL with ketamine). In line with the study of Daskapoulou [9] almost three-quarter of MSM engaging in chemsex reported sober sex in the past month, indicating that dependency might be only an issue for a minority. As Holt [24] stated, not all drug use has to be automatically framed as problematic. Findings from other studies showed that many men who engaged in chemsex successfully managed their drug use and found it enjoyable. Nevertheless, the risk of addiction related to the use of a cheap depressant such as GHB/GBL [25] needs awareness among health services and emphasizes the need of identifying those who need help.

Concerning the STI risk related to the specific drugs used during sex, our study shows that XTC/MDMA, GHB/GBL, and cocaine were associated with STI after adjustment for sociodemographic characteristics and non-drug use sexual history. However, the widespread pattern of polydrug use among MSM makes if difficult to relate findings to one specific drug. Our study shows that the use of at least three drugs included in the broad definition of chemsex was the only independently associated determinant for STI in the final model. This finding confirms results from other studies in both HIV-positive [9, 26] and HIV-negative MSM [27, 28], in which strong associations were found between polydrug use and sexual risk behaviour. We looked further into the concept of polydrug use and noted that among MSM using three of more drugs, 91% used XTC/MDMA and GHB/GBL. We found no predominant combinations of three or four specific drugs. It is noteworthy that 98% of men using one of the typical chemsex drugs (crystal meth, GHB/GBL or mephedrone) also used other drugs. Because the use of other drugs than the typical chemsex drugs was associated with STI and men using the typical chemsex drugs always used other drugs as well, we would recommend STI prevention and care strategies to broaden their definition of chemsex. As recommended in the British Association for Sexual Health and HIV (BASHH) guidelines [29], health care providers should be aware of trends in drug use in their locality and their effects and STI clinics should incorporate these trends into sexual history taking for clients attending for STI screening Currently, pre-exposure prophylaxis (PrEP) is becoming more widely available for MSM in most Western countries. A study in Amsterdam found a PrEP use prevalence among MSM of 7% in 2017, of which two-third was study-provided PrEP [30]. During the data collection period in the current study, we expect that only a small minority of our participants already used PrEP. PrEP was not yet covered by health insurances in the Netherlands and there were no PrEP studies offering free PrEP to our study population. However, PrEP use among MSM is expected to increase during the upcoming years [31]. A recent study in the Netherlands showed that over the first six months after initiation of PrEP, an increase in condomless anal sex with casual partners was observed [32]. Therefore, we recommend future studies to include PrEP use when assessing the relationship between chemsex and STI.

To our best knowledge, this is the first study on chemsex among MSM outside major cities in the Netherlands. We included a wide variety of drugs next to the three typical chemsex drugs, which allowed us to describe drug use patterns and their associations with STI. Another strength of our study was the use of STI test results instead of self-reported STI. However, some limitations are worth noting. One general limitation of our study was the cross-sectional design, in which causality and temporality cannot be determined, making it impossible to investigate the causal mechanisms through which drug use and STI operate. A limitation for the assessment of the prevalence of chemsex in MSM visiting the STI clinic outside the major cities in the Netherlands, was the risk of sampling bias. Only one-third of MSM visiting the STI clinic during the study period was asked to participate. However, several precautionary measures were taken to decrease the risk of systematic sampling bias. STI nurses were explicitly instructed to recruit MSM who visited the STI clinic regardless of their drug using and STI status. The reason that nurses did not recruit all MSM who visited the clinic was mostly for random reasons (e.g. too short time, forgotten to ask). Indeed, no differences in age, educational level, ethnicity, and STI positivity were found between MSM who were recruited and MSM who were not recruited. Furthermore, the invitation for the questionnaire that was sent to the recruited MSM was developed to be appealing also for MSM who did not use drugs and questionnaires were available in Dutch and English. The response rate on the questionnaire was high (70%). We compared MSM who completed the questionnaire with MSM who were recruited but not completed the questionnaire. We showed that age, ethnicity, and STI positivity were comparable between those groups, but MSM who completed the questionnaire were more often higher educated. When comparing participants, MSM who completed the questionnaire, with all non-participants, MSM who were not recruited or did not complete the questionnaire, we showed that age and STI positivity were comparable but participants were slightly higher educated and more often had a western nationality. Educational level and ethnicity were not associated with drug use during sex in our study. Therefore, we expect that our estimated proportion of MSM engaging in drug use during sex is representative for the high-risk MSM who are served by STI clinics in the Netherlands. A limitation for the assessment of associations between the use of specific drugs during sex and STI, was that some drugs were used by too few MSM to calculate associations. In previous studies, the use of crystal meth or mephedrone has been associated with STI [33–35], but we were unable to test these associations. However, we show that MSM using these drugs always use other drugs as well, indicating that the STI risk of using these drugs may have been reflected in the association between number of drugs used and STI.

## Conclusion

This study showed that drug use during sex is also prevalent among MSM visiting the STI clinic outside the major cities in the Netherlands. This indicates that STI clinics in both urban and non-urban areas should be aware of and be informed on this phenomenon. MSM using one of the typical chemsex drugs always use other drugs as well, and especially this behaviour—polydrug use—was associated with STI. This indicates that both STI prevention and drug harm reduction strategies might need to broaden the chemsex definition and include interactions between drugs.

## Supporting information

S1 File List of all drug types used before or during sex in preceding six months asked in our study questionnaire (both standard used names (bolt) and street names). (DOCX)

S2 File Questionnaire in both Dutch and English. (DOCX)
