# Supplementary material for: Chemsex among men who have sex with men living outside major cities and associations with sexually transmitted infections: A cross-sectional study in the Netherlands
Source: PLoS One. 2019 May 14;14(5):e0216732. doi: 10.1371/journal.pone.0216732 (PMC6516639; doi:10.1371/journal.pone.0216732)
Supplement: S1 File — (DOCX) [file pone.0216732.s001.docx]

**S1. List of all drug types used before or during sex in preceding six months asked in our study questionnaire (both standard used names (bolt) and street names)**

| **Basecoke**, Crack |
| --- |
| **Cannabis**, Hashish, Weed, Marijuana |
| **Cocaine,** Coke, Charlie, Blow, Snow, White |
| **Crystal meth**, Tina, Ice, Crystal, T, Shabu, Yaba, Shista, Glass |
| **GHB or GBL**, G, Liquid Ecstasy, Gina |
| **Heroin**, Smack, H, Brown, Sugar, Chiva, Chiba |
| **Ketamine**, K, Special K, Keta, Ket, Vitamin K, Donkey Dust |
| **Laughing gas**, Nitrous Oxide |
| **LSD,** Acid |
| **Mephedrone,** Meow Meow, 4-MCC, M-Cat, Miaow, Plant food, MMC-4, Bubble |
| **XTC or MDMA,** M, Molly, Ecstasy, Pill, Candy |
| **MXE,** Methoxet amine, Mexxxy, Roflocptr |
| **Naphyrone,** NRG |
| **Magic mushrooms** |
| **Poppers,** Nitrite Inhalants |
| **Ritalin,** Concerta, Dexamphetamine (not used as medication for ADD/ADHD) |
| **Speed,** Amphetamine, Pep |
| **Designer drugs (2-CB, 3 MMC, 4-FA, 4-MEC)** |
