# Supplementary material for: Chemsex among men who have sex with men living outside major cities and associations with sexually transmitted infections: A cross-sectional study in the Netherlands
Source: PLoS One. 2019 May 14;14(5):e0216732. doi: 10.1371/journal.pone.0216732 (PMC6516639; doi:10.1371/journal.pone.0216732)
Supplement: S2 File — (DOCX) [file pone.0216732.s002.docx]

**S2 Questionnaire in both Dutch and English**

Questions in Dutch

1. Heb je ooit wel eens drugs gebruikt? Dit gebruik hoeft niet samen te hangen met seks, dit kan ook bijvoorbeeld tijdens een festival zijn geweest.

- Ja
- Nee *>> Vraag 11*

1. Welke drugs heb je ooit gebruikt? Geef per drug aan of je deze ooit hebt gebruikt (ja of nee).

|  | **Ja** | **Nee** |
| --- | --- | --- |
| **Basecoke**, Crack |  |  |
| **Cannabis**, Hasj, Wiet, Marijuana |  |  |
| **Crystal meth**, Tina, Ice |  |  |
| **Cocaine**, Coke, Charlie, Blow, Snow, White |  |  |
| **GBL**, G, G-tje, Buisje, Tante Gea, Liquid Ecstasy |  |  |
| **GHB**, G, G-tje, Buisje, Tante Gea, Liquid Ecstasy |  |  |
| **Heroïne,** H, Smack, Chiba, Chiva, Bruin |  |  |
| **Ketamine,** K, Special K, Keta, Ket, Vitamin K |  |  |
| **Lachgas** |  |  |
| **LSD** |  |  |
| **Mephedrone,** Meow Meow, 4-MCC, M-Cat, Drone, Miaow, Plant food |  |  |
| **MDMA,** M, Molly |  |  |
| **MXE,** Mexxxy, Roflocptr |  |  |
| **Naphyrone,** NRG |  |  |
| **Paddo’s, truffels** |  |  |
| **Poppers** |  |  |
| **Ritalin, Concerta,** Dexamphetamine (niet als medicatie tegen ADD/ADHD) |  |  |
| **Speed,** Amphetamine, Pep |  |  |
| **XTC,** Ecstasy, Pil, Candy |  |  |
| **2-CB** |  |  |
| **3 MMC** |  |  |
| **4-FA,** 4-FMP, 4 Fluor, 4F, Flava |  |  |
| **Andere drugs** |  |  |

1. Je hebt net aangegeven dat je een andere drug hebt gebruikt dan in de tabel is aangegeven. Welke drug(s) zijn dit? Je kunt één, twee of drie andere namen van drugs invoeren.

- Naam andere drug: *Open*
- Naam andere drug: *Open*
- Naam andere drug: *Open*

1. Heb je wel eens drugs voor of tijdens seks gebruikt?

In deze vragenlijst bedoelen we met seks elke vorm van seksuele activiteit: anale seks

(kont), vaginale seks (vagina), manuele seks (handen) orale seks (mond) en seks met

speeltjes.

- - Ja
  - Nee *>> Vraag 11*

1. Hoe vaak heb je de volgende drugs gebruikt voor of tijdens seks in de afgelopen zes maanden?

*Als je een andere drug hebt gebruikt dan is aangegeven in onderstaande tabel, klik dan terug naar vraag 2 om deze drug aan te kruisen. Linksboven in de hoek van het scherm kun je terug klikken naar de vorige vraag.*

*>>Drugs die zijn aangeklikt bij vraag 2 worden weergegeven*

|  | 4 of meer keer per week | 2-3 keer per week | 2-4 keer per maand | 1 keer per maand of minder | Niet in de afgelopen zes maanden | Niet voor of tijdens seks |
| --- | --- | --- | --- | --- | --- | --- |
| Basecoke, Crack | 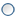 | 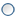 | 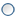 | 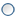 | 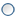 | 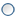 |
| Cannabis, Hasj, Wiet, Marijuana | 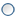 | 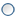 | 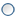 | 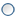 | 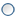 | 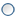 |
| Crystal meth, Tina, Ice, Crystal, T, Shabu, Yaba, Shista | 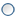 | 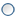 | 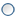 | 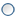 | 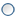 | 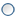 |
| Cocaïne, Coke, Charlie, Blow, Snow, White | 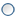 | 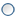 | 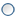 | 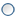 | 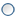 | 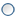 |
| GBL, G, G-tje, Buisje, Tante Gea, Liquid Ecstasy | 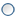 | 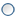 | 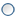 | 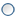 | 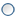 | 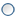 |
| GHB, G, G-tje, Buisje, Tante Gea, Liquid Ecstasy | 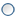 | 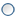 | 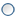 | 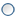 | 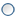 | 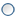 |
| Heroïne, H, Smack, Chiba, Chiva, Bruin | 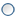 | 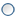 | 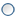 | 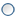 | 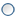 | 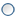 |
| Ketamine, K, Special K, Keta, Ket, Vitamin K | 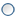 | 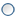 | 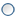 | 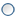 | 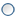 | 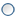 |
| Lachgas | 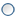 | 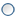 | 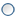 | 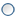 | 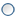 | 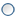 |
| LSD | 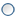 | 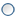 | 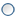 | 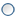 | 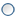 | 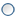 |
| Mephedrone, Meow Meow, 4-MCC, M-Cat, Drone, Miaow, Plant Food, MMC-4 | 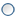 | 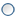 | 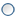 | 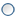 | 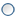 | 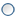 |
| MDMA, M, Molly | 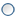 | 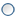 | 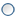 | 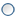 | 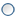 | 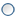 |
| MXE, Mexxxy, Rofloctptr | 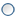 | 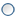 | 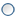 | 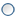 | 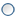 | 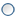 |
| Naphyrone, NRG | 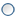 | 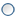 | 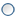 | 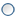 | 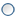 | 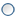 |
| Paddo‘s | 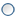 | 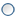 | 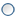 | 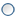 | 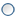 | 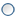 |
| Poppers | 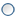 | 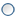 | 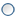 | 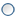 | 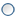 | 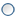 |
| Ritalin, Concerta, Dexamfetamine (niet als medicijn voor ADD/ADHD) | 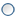 | 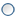 | 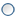 | 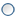 |  |  |
| Speed, Amfetamine, Pep |  |  |  |  |  |  |
| XTC, Ecstasy, X, Pill, Candy |  |  |  |  |  |  |
| 2-CB |  |  |  |  |  |  |
| 3 MMC |  |  |  |  |  |  |
| 4-FA, 4-FMP, Fluor, 4-F, F, Flava |  |  |  |  |  |  |
| 4-MEC |  |  |  |  |  |  |
| [{Drug1}] |  |  |  |  |  |  |
| [{Drug2}] |  |  |  |  |  |  |
| [{Drug3}] |  |  |  |  |  |  |

1. De volgende vragen gaan over het combineren van verschillende drugs. Met drugs combineren bedoelen wij verschillende drugs tegelijk of (vlak) na elkaar gebruiken.
2. Combineer je wel eens verschillende drugs voor of tijdens seks?

- Ja, geef aan welke combinatie je het meest gebruikt *Open*
- Nee *>> Vraag 9*

1. Hoe vaak heb je verschillende drugs gecombineerd voor of tijdens seks in de afgelopen zes maanden?

- 4 of meer keer per week
- 2-3 keer per week
- 2-4 keer per maand
- 1 keer per maand of minder
- Niet in de afgelopen zes maanden

1. Hoe vaak heb je alcohol en drugs gecombineerd voor of tijdens seks in de afgelopen zes maanden?

- 4 of meer keer per week
- 2-3 keer per week
- 2-4 keer per maand
- 1 keer per maand of minder
- Niet in de afgelopen zes maanden *>> Vraag 11*
- Nooit *>> Vraag 11*

1. Hoeveel glazen alcohol heb je de laatste keer gedronken toen je alcohol en drugs combineerde voor of tijdens seks? Vul een getal in. Dit mag een schatting zijn.

*Open*

1. Welke van onderstaande middelen heb je wel eens gebruikt in de afgelopen zes maanden? Je kunt één of meerdere antwoorden aankruisen.

- **Androskat**
- **Erectiemiddelen**, bijvoorbeeld Kamagra, Viagra, Cialis, Sildenafil, Vardenafil
- **Slaap- of kalmeringsmiddelen**, bijvoorbeeld Benzo’s, Morphine, Valium, Oxazepam, Temazepam, Zolpidem
- **Testosteron,** Anabole Steroïden, DHEA
- **Nitraten,** nitroglycerine, isosorbidenitraat, isosorbidemononitraat
- **Geen van bovenstaande middelen**

1. Hoe heb je drugs of andere middelen genomen in de afgelopen zes maanden? Je kunt één of meer antwoorden aankruisen.

*Deze vraag wordt alleen gesteld aan deelnemers die drugs en/of middelen hebben aangekruist.*

- Slikken (pil, drankje, bommetje (poeder in vloeitje))
- Laten smelten onder mijn tong
- Snuiven
- Roken
- Anaal (kont) inbrengen zonder spuit
- Spuiten in mijn kont zonder naald (booty bumping)
- Spuiten met een naald (slammen) door mijzelf of iemand anders
- Op een andere manier, namelijk *Open*

1. Heeft iemand anders dan jijzelf bij jou ooit drugs of middelen ingespoten met een naald (geslamd)?

- Ja
- Nee
- Weet ik niet meer

1. Deel je wel eens naalden met anderen?

*Deze vraag wordt alleen gesteld aan deelnemers die bij vraag 12 hebben aangekruist:*

*Spuiten met een naald (slammen) door mijzelf of iemand anders*

- Ja
- Nee
- Weet ik niet meer

1. Deel je wel eens spuiten zonder naald met anderen?

*Deze vraag wordt alleen gesteld aan deelnemers die bij vraag 12 hebben aangekruist:*

*Spuiten in mijn kont zonder naald (booty bumping)*

- Ja
- Nee
- Weet ik niet meer

1. Deel je wel eens materialen die je gebruikt om drugs te snuiven (bijvoorbeeld snuifbuisjes, opgerolde bankbiljetten)?

*Deze vraag wordt alleen gesteld aan deelnemers die bij vraag 12 hebben aangekruist:*

*Snuiven*

- Ja
- Nee
- Weet ik niet meer

1. Met hoeveel partners heb je meestal seks per keer als je drugs gebruikt?

- 1
- 2 of 3
- 4 of meer
- Meer dan 10
- Dat kan ik me niet herinneren

1. Met wie heb je seks wanneer je drugs gebruikt? Je kunt één of meer antwoorden aankruisen.

- Vaste partner (relatie)
- Vaste sekspartner(s) (fuck-buddy)
- Losse sekspartner(s) (je weet zijn naam)
- Groepsseks
- Klanten
- Vrienden
- Losse sekspartner(s) (je weet zijn naam niet)
- Vrouw
- Anders, namelijk *Open*

1. Hoe lang heb je meestal seks als je drugs gebruikt (met pauzes)?

- Eén uur of minder
- Meerdere uren
- Een nacht lang (12 uur)
- Ruim een etmaal (24 uur)
- Meerdere dagen achter elkaar

1. Welke seks had je toen je drugs gebruikte in het afgelopen jaar? Je kunt één of meer antwoorden geven?

- Aftrekken
- Ik pijpte hem
- Hij pijpte mij
- Zijn penis in mijn kont **met condoom** (anale seks ontvangend, bottom)
- Zijn penis in mijn kont **zonder condoom** (anale seks ontvangend, bottom)
- Mijn penis in zijn kont **met condoom** (anale seks gevend, top)
- Mijn penis in zijn kont **zonder condoom** (anale seks gevend, top)
- Ik likte zijn kont (rimmen)
- Zijn vuist in mijn kost (fisten)
- Speeltjes in mijn kont zonder condoom
- Ik weet het niet meer precies
- Anders, namelijk *Open*

Questions in English

1. Have you ever used drugs? This also includes recreational drug use, for example during a festival.

- Yes
- No *>> Question 11*

1. Which drugs have you ever used? Please indicate for every drug if you ever used it (yes or no).

|  | **Yes** | **No** |
| --- | --- | --- |
| **Basecoke**, Crack |  |  |
| **Cannabis**, Hashish, Weed, Marijuana |  |  |
| **Crystal meth**, Tina, Ice |  |  |
| **Cocaine**, Coke, Charlie, Blow, Snow, White |  |  |
| **GBL**, G, Gina, Liquid Ecstasy |  |  |
| **GHB**, G, Gina, Liquid Ecstasy |  |  |
| **Heroin,** H, Smack, Chiba, Chiva, Brown |  |  |
| **Ketamine,** K, Special K, Keta, Ket, Vitamin K |  |  |
| **Laughing gas** |  |  |
| **LSD,** Acid |  |  |
| **Mephedrone,** Meow Meow, 4-MCC, M-Cat, Drone, Miaow, Plant food |  |  |
| **MDMA,** M, Molly |  |  |
| **MXE,** Mexxxy, Roflocptr |  |  |
| **Naphyrone,** NRG |  |  |
| **Magic mushrooms** |  |  |
| **Poppers** |  |  |
| **Ritalin, Concerta,** Dexamphetamine (not used as medication for ADHD/ADD) |  |  |
| **Speed,** Amphetamine, Pep |  |  |
| **XTC,** Ecstasy, Pil, Candy |  |  |
| **2-CB** |  |  |
| **3 MMC** |  |  |
| **4-FA,** 4-FMP, 4 Fluor, 4F, Flava |  |  |
| **Other drug(s)** |  |  |

1. You just indicated that you used other drug(s). Please write down the name of the other drug(s). The maximum number is 3.

- Name other drug: *Open*
- Name other drug: *Open*
- Name other drug: *Open*

1. Have you ever used drugs before or during any kind of sex?

*In this questionnaire, sex is considered to be any kind of sexual activity: anal sex*

*(butt), vaginal sex (vagina), oral sex (mouth), manual sex (hands), and sex with toys.*

- - Yes
  - No *>> Question 11*

1. How often have you used the following drugs before or during sex in the past six months?

*If you used other drugs than indicated below, click back to question 2 to check this drug (in the upper left corner of the screen you can click back to the previous question).*

*>>Drugs that are checked in question 2 are displayed.*

|  | 4 or more times per week | 2-3 times per week | 2-4 times per month | 1 time per month or less | Not in the past six months | Not before or during sex |
| --- | --- | --- | --- | --- | --- | --- |
| Basecoke, Crack |  |  |  |  |  |  |
| Cannabis, Hashish, Weed, Marijuana |  |  |  |  |  |  |
| Crystal meth, Tina, Ice, Crystal, T, Shabu, Yaba, Shista |  |  |  |  |  |  |
| Cocaine, Coke, Charlie, Blow, Snow, White |  |  |  |  |  |  |
| GBL, G, Gina, Liquid Ecstasy |  |  |  |  |  |  |
| GHB, G, Gina,, Liquid Ecstasy |  |  |  |  |  |  |
| Heroin, H, Smack, Chiba, Chiva, Bruin |  |  |  |  |  |  |
| Ketamine, K, Special K, Keta, Ket, Vitamin K |  |  |  |  |  |  |
| Laughing gas |  |  |  |  |  |  |
| LSD, Acid |  |  |  |  |  |  |
| Mephedrone, Meow Meow, 4-MCC, M-Cat, Drone, Miaow, Plant Food, MMC-4 |  |  |  |  |  |  |
| MDMA, M, Molly |  |  |  |  |  |  |
| MXE, Mexxxy, Rofloctptr |  |  |  |  |  |  |
| Naphyrone, NRG |  |  |  |  |  |  |
| Magic mushrooms |  |  |  |  |  |  |
| Poppers |  |  |  |  |  |  |
| Ritalin, Concerta, Dexamfetamine (no used as medication for ADHD/ADD) |  |  |  |  |  |  |
| Speed, Amfetamine, Pep |  |  |  |  |  |  |
| XTC, Ecstasy, X, Pill, Candy |  |  |  |  |  |  |
| 2-CB |  |  |  |  |  |  |
| 3 MMC |  |  |  |  |  |  |
| 4-FA, 4-FMP, Fluor, 4-F, F, Flava |  |  |  |  |  |  |
| 4-MEC |  |  |  |  |  |  |
| [{Drug1}] |  |  |  |  |  |  |
| [{Drug2}] |  |  |  |  |  |  |
| [{Drug3}] |  |  |  |  |  |  |

1. The next few questions will be about combining different drugs. By combining drugs we mean using different drugs simultaneously or (immediately) after each other.
2. Have you ever combined different drugs before or during sex?

- Yes, please indicate which combination you use most often *Open*
- No *>> Question 9*

1. How often have you combined different drugs before or during sex in the past six months?

- 4 or more times per week
- 2-3 times per week
- 2-4 keer per month
- 1 time per month or less
- Not in the past six months

1. How often have you combined alcohol and drugs before or during sex in the past six months?

- 4 or more times per week
- 2-3 times per week
- 2-4 times per month
- 1 time per month or less
- Not in the past six months *>> Question 11*
- Never *>> Question 11*

1. How many glasses of alcohol did you consume the last time you combined alcohol and drugs before or during sex? Please write down a number. It can be an estimation. *Open*
2. Which of the following substances have you used in the past six months? Please select all that apply.

- **Androskat**
- **Substances tht help to keep an erection**, for example Kamagra, Viagra, Cialis, Sildenafil, Vardenafil
- **Sleep medicines or sedatives**, for example Benzos, Morphine, Valium, Oxazepam, Temazepam, Zolpidem
- **Testosterone,** Anabolic steroids, DHEA
- **Nitrates,** nitroglycerin, isosorbide nitrate, isosorbide mononitrate
- **None of the above substances**

1. How did you administer drugs or substances you used before or during sex in the past six months?

*This question is shown only to participants who checked drugs or substances.*

- Swallow (pill, liquid, bomb (powder in a paper))
- Melt under my tongue
- Snort/inhale
- Smoke
- Rectal administration without a syringe
- Rectal administration with a syringe without a needle (booty bumping)
- Inject (slamming) by myself or somebody else
- Other way, please specify *Open*

1. Has someone else ever injected (slammed) drugs into your body?

- Yes
- No
- I do not remember

1. Have you ever shared needles with others?

*This question is shown only to participants who checked (in question 12):*

*Inject (slamming) by myself or somebody else*

- Yes
- No
- I do not remember

1. Have you ever shared syringes without needles with others?

*This question is shown only to participants who checked (in question 12):*

*Rectal administration with a syringe without a needle (booty bumping)*

- Yes
- No
- I do not remember

1. Have you ever shared materials to snort drugs with others (for example snort tubes, rolled banknotes)?

*This question is shown only to participants who checked (in question 12):*

*Snort/inhale*

- Yes
- No
- I do not remember

1. How many different steady male partners have you had usually when you used drugs before or during sex?

- 1
- 2 or 3
- 4 or more
- More than 10
- I do not remember

1. With whom did you have sex with when you used drugs? Please select all that apply.

- Regular partner (relationship)
- Regular sex partner (fuck-buddy)
- Casual sex partner(s) (you know their name)
- Group sex
- Customers
- Friends
- Casual sex partner(s) (you do not know their name)
- Woman
- Other, please specify *Open*

1. How long do you normally have sex when you use drugs (including breaks)?

- One hour or less
- Several hours
- One night (12 hours)
- More than a day (24 hours)
- Several days in a row

1. What kind of sex did you have when you used drugs in the past six months? Please select all that apply.

- Jerk off (wanking)
- I gave him a blow-job
- He gave me a blow-job
- His penis in my anus **with a condom** (receptive anal sex, bottom)
- His penis in my anus **without a condom** (receptive anal sex, bottom)
- My penis in his anus **with a condom** (insertive anal sex, top)
- My penis in his anus **without a condom** (insertive anal sex, top)
- I licked his anus (rimming)
- His fist in my anus (fisting receptive)
- Toys in my anus without a condom
- I do not remember
- Other, please specify *Open*
